# Electrocatalytic Ammonia Oxidation with Coordinatively Saturated Ruthenium Catalyst

**DOI:** 10.1021/acs.inorgchem.5c02418

**Published:** 2025-07-01

**Authors:** Chuan-Pin Chen, Oluwafemi Abubakar, Xiaoyin Zhang, Thomas W. Hamann

**Affiliations:** Department of Chemistry, 3078 Michigan State University, 578 S Shaw Ln, East Lansing, Michigan 48824, United States

## Abstract

This communication describes the investigation of a coordinatively saturated complex [Ru­(tpy)­(dmabpy)­Cl]^+^ ([Ru­(Cl)]^+^) as an ammonia oxidation catalyst. Cyclic voltammetry measurements show an ideal S-shaped wave, indicating total catalysis conditions with a *k*_obs_ (TOF_max_) of 9360 h^–1^. The reaction was found to be first-order in [Ru­(Cl)]^+^ and third-order in [NH_3_]. Stoichiometric reactions of the one-electron oxidized species, [Ru­(Cl)]^2+^, were monitored following the addition of ^15^NH_3_ using ^1^H and ^15^N NMR spectroscopy. These experiments showed that the chloride-coordinated complex rapidly converts NH_3_ to N_2_. NMR spectroscopy and electrochemistry results definitively show that NH_3_ does not substitute the Cl^–^ ligand to form the previously reported [Ru­(NH_3_)]^2+^ catalyst which operates by a different mechanistic pathway. Taken together these results indicate outersphere electron transfer mediated ammonia oxidation reaction.

## Graphical abstract

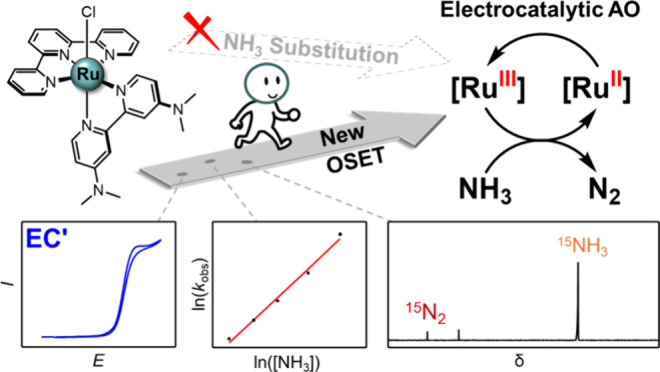

Ammonia (NH_3_) is rapidly becoming recognized as a promising medium to store and distribute renewable hydrogen (H_2_) and enable the realization of the hydrogen economy.^1–3^ To release the hydrogen stored in ammonia, the hydrogen evolution reaction (HER) must be coupled with the ammonia oxidation reaction (AOR). While there is a long history of developing catalysts competent to carry out the HER, the AOR has only recently received attention. Our group reported the first example of a homogeneous electrocatalyst for ammonia oxidation under mild conditions; [Ru­(tpy)­(dmabpy)­NH_3_]^2+^ (Ruthenium = Ru, tpy = terpyridine; dmabpy = 4,4′-bis­(dimethylamino)-2,2′-bipyridine), [Ru­(NH_3_)]^2+^, the structure of which is shown in Chart 1.^4^ The electrocatalytic ammonia oxidation reaction with [Ru­(NH_3_)]^2+^ is triggered by electrochemical oxidation of Ru^II^ to Ru^III^ followed by deprotonation of the coordinated ammine to form [Ru­(NH_2_)]^2+^, which undergoes a redox disproportionation reaction to regenerate [Ru­(NH_3_)]^2+^ and form the [Ru­(NH)]^2+^ intermediate species. This imido intermediate can be attacked by NH_3_ to form a hydrazine complex which is ultimately oxidized to N_2_.^4^ Stoichiometric reaction of the one-electron oxidized [Ru­(NH_3_)]^3+^ complex with NH_3_ and noncoordinating bases such as collidine showed that the [Ru­(NH)]^2+^ aminyl intermediate undergoes a sequential disproportionation reaction to produce a nitride that rapidly couples to make a dinitrogen-bridged complex, [Ru­(N≡N)­Ru]^4+^. ^15^N NMR spectroscopy measurements of the mixture of [Ru­(NH_3_)]^3+^ with NH_3_ indicated both mechanisms above are operative and contribute to the overall electrocatalytic AOR.^5^ Recently, Roithová and Zhou integrated voltammetry with electrospray ionization mass spectrometry (VESI-MS) and photodissociation spectroscopy to detect elusive intermediates during electrocatalysis with [Ru­(NH_3_)]^2+^.^6^ They detected Ru^III^ aminyl and Ru^IV^ imido complexes as well as hydrazine and diazene intermediates. Based on these data, they proposed a third mechanism of N–N bond formation by homocoupling of aminyl intermediates to form [Ru­(NH_2_NH_2_)­Ru]^4+^. Thus, three distinct pathways of conversion of NH_3_ to N_2_ operate in parallel, all of which involve bimolecular reactions of redox disproportionation or homocoupling, with [Ru­(NH_3_)]^3+^. Similar reaction pathways have been reported for other AOR catalysts.^7–11^

**Chart 1:**
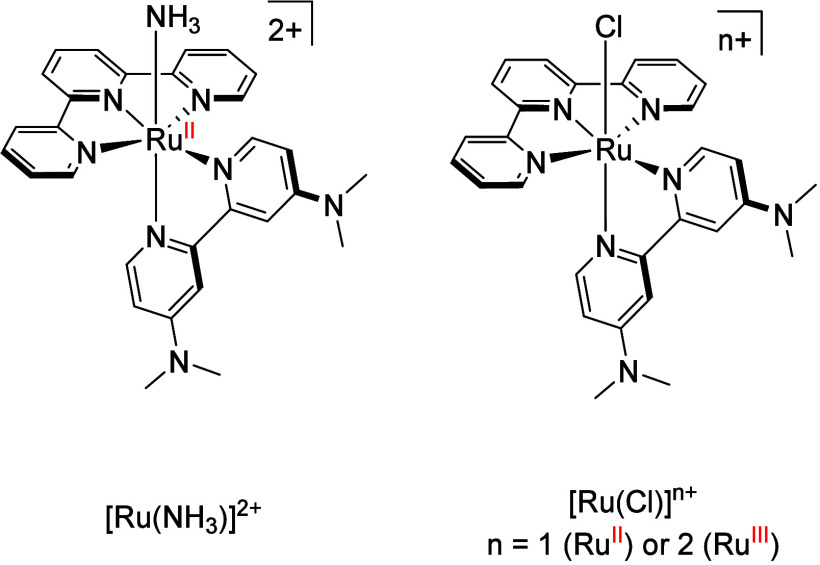
Chemical Structures of the Ruthenium Complexes Discussed in This Letter

Tilley and co-workers reported a novel host–guest complex for ammonia oxidation using an analogue of [Ru­(NH_3_)]^2+^, with an adamantyl substituent added to the terpyridine ligand to serve as a guest moiety, [Ru­(dmabpy)­(tpada)­Cl]­(PF_6_) (tpada = 4′-(adamantan-1-yl)-2,2’:6′,2″-terpyridine).^12^ Due to the strong binding interaction of the adamantyl group with a β-cyclodextrin host species, the Ru complex anchors to a β-cyclodextrin-modified electrode, forming a heterogeneous host–guest (HG) complex on the electrode surface. They showed that the electrocatalytic AOR is viable with the heterogenized catalyst, which was attributed to initial ligand exchange of coordinated chloride by NH_3_ in a saturated ammonia solution. Interestingly, the immobilization of the catalyst should prevent redox disproportionation or other bimolecular reaction pathways, suggesting yet another mechanism of AOR with [Ru­(NH_3_)]^2+^. This possibility motivated us to investigate the behavior of nonammine coordinated complexes, such as [Ru­(Cl)]^+/2+^ (Chart 1) as a catalyst, or precatalyst, to drive the AOR and investigate the reaction mechanism.

[Ru­(Cl)]Cl was prepared following previously published methods.^4,13,14^ Ion exchange was performed to replace the chloride counterion with PF_6_^–^ to obtain [Ru­(Cl)]­(PF_6_). The strong one-electron oxidant tris­(4-bromophenyl)­ammoniumyl hexafluorophosphate ([NAr_3_]­[PF_6_]), “magic blue”, was used to oxidize the Ru^II^ metal center to form [Ru­(Cl)]­(PF_6_)_2_.^5^ Detailed procedures and characterization of complexes are provided in the Supporting Information (Figures S1–S7).

Cyclic voltammograms (CV) of [Ru­(Cl)]^+^ were measured in dry MeCN containing 0.1 M of ammonium trifluoromethanesulfonate (NH_4_OTf) as a supporting electrolyte using a boron-doped diamond (BDD) working electrode, platinum mesh counter electrode, and a homemade silver chloride/silver reference electrode. Potentials were corrected to the ferrocenium/ferrocene redox couple, which was measured as an external standard. The scan rate (ν) dependent CVs exhibit increasing peak currents with increasing scan rates showing diffusion-controlled behavior (Figures S8 and S9).^15^ Addition of ammonia to saturation (1.32 M) results in a significantly enhanced anodic current which plateaus at positive potentials with concomitant loss of the cathodic return wave when measured at ν = 0.01 V/s, indicating pure kinetic electrocatalytic behavior (Figure 1).^16,17^ This finding is consistent with the previous report by Tilley.^12^ We note, this electrocatalytic response is quite different from our previously reported measurements of [Ru­(NH_3_)]^2+^, however, or any other reported AOR catalyst.^4^

**Figure 1:**
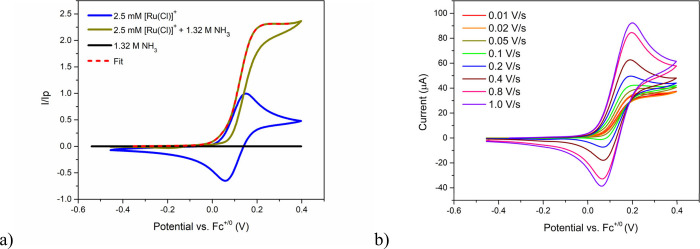
a) Cyclic voltammograms of 1.32 M NH_3_ (black), 2.5 mM [Ru­(Cl)]^+^ before (blue) and after (mustard) adding 1.32 M NH_3_ at a scan rate 0.05 V/s in MeCN solution containing 0.1 M NH_4_OTf electrolyte. Currents are normalized to the anodic peak current of [Ru­(Cl)]^+^ in the absence of catalysis. Also shown is the fitted curve to eq 2 (red dashed). b) Cyclic voltammograms of 2.5 mM [Ru­(Cl)]^+^ with 1.32 M NH_3_ measured with scan rates from 0.01 – 1.0 V/s.

The CVs show scan rate independent, ideal ‘S’ shaped current responses at low scan rates from 0.01 – 0.05 V/s consistent with total catalysis regime.^16,18^ Using the approximation that the reaction follows an EC′ mechanism according to

$$E:,Ru^{II}\overset{k_{E}}{⇌}Ru^{III}+e^{-}$$

$$C^{\prime }:,Ru^{III}+S\overset{k_{C}}{\to }Ru^{II}+P$$

The pseudo first-order rate constant, *k*_obs_ = *k*_C_[S], determines the rate of the catalytic chemical step, which can be determined from the catalytic plateau current, *i*_c_, according to

1 $$i_{c}=nFAC_{Ru}^{0}\sqrt{Dk_{obs}}$$

where *n* is the number of electrons transferred, *F* is faradays constant, *A* is the electrode area and *C*_*Ru*_^0^ is the bulk Ru catalyst concentration. The *i*_c_ increased linearly with *C*_*Ru*_^0^ from 1.0 – 5.0 mM as expected from eq 1, indicating first order behavior in catalyst, shown in Figure S10, but only marginally increases at 10 mM. This behavior is reproducible and indicates the catalysis saturates at high loadings for reasons that are not yet clear. Further investigations therefore employed 2.5 mM of [Ru­(Cl)]^+^. To avoid errors involved with *A*, *C*_*Ru*_^0^ and *D*, the ratio of catalytic current and peak current in the absence of substrate can be used to determine *k*_obs_ at a given scan rate according to^16,19,20^

2 $$\frac{i}{i_{p,a}}=\frac{2.24\sqrt{\left(\frac{RT}{fv}\right)k_{obs}}}{1+\exp\left|\frac{f}{RT}\left(E_{cat}-E\right)\right|}$$

A nonlinear least-squares fit result of eq 2 to the forward catalytic wave is shown in Figure 1a, which produced *k*_obs_ = 2.06 ± 0.01 s^–1^ and *E*_*cat*_ = 0.116 V. This was reproduced multiple times, using only the low scan rates where *i*_c_ is constant, with an average and standard deviation of 2.6 ± 1 s^–1^ (9,360 h^–1^). The concentration of NH_3_ was varied by dilution of a NH_3_ saturated electrolyte solution containing 2.5 mM of [Ru­(Cl)]^+^ and 0.1 M NH_4_OTf with a solution containing 2.5 mM of [Ru­(Cl)]^+^ and 0.1 M NH_4_OTf without NH_3_, so only the concentration of NH_3_ changes in a controllable fashion. The *i*_c_ increases approximately linearly with [NH_3_] (Figure S11). Given that *k*_obs_ = *k*_C_[NH_3_]^α^, where α is the reaction order with respect to NH_3_, a plot of two independent sets of titration results of ln­(*k*_obs_) vs ln­([NH_3_]) was made, Figure S11, which yielded a straight line with a slope of 2.9 ± 0.4. This unusual third order reaction is similar to the reported order of 2.55 in [NH_3_] reported for AOR with ferrocenium, suggesting the same transition state and reaction mechanism.^21,22^

Increasing the scan rate above 0.1 V/s for [Ru­(Cl)]^+^ solutions containing 1.3 M NH_3_ results in the emergence of an anodic peak and return cathodic peak, which becomes more prominent with increasing scan rate and appears quasi-reversible at scan rates of ∼1 V/s (Figure 1b). Similar behavior is also observed at lower concentrations of NH_3_. These phenomena are attributed to the electrochemical reduction of Ru^III^ to Ru^II^ (−*k*_E_) outcompeting the relatively slow rate of ammonia oxidation by the oxidized catalyst (*k*_C_). The ideal s-shaped catalytic response seen in Figure 1 allows precise determination of the overpotential, which is the difference between the standard thermodynamic potential of ammonia oxidation and the potential applied to produce a given rate. The standard potential, $E_{N_{2}/{NH}_{3}}^{◦}$ , in MeCN is –0.94 V vs Fc^+/0^.^23^ Given the 10-fold excess of [NH_3_] relative to [NH_4_^+^], the reversible potential is –1.00 V vs Fc^+/0^.^9,23^ The potential applied to generate 1/2 the plateau current, *E*_cat_,^20^ was found to be 0.116 V vs Fc^+/0^ from the fit shown in Figure 1a to eq 2, thus, the overpotential is 1.116 V.

Prior reports of the related catalysts with a Cl^–^ ligand, suggested that catalysis is initiated following substitution of Cl^–^ by NH_3_ to generate the active NH_3_ bound catalyst which enters a catalytic cycle such as described above.^12,24,25^ Chloride can be a kinetically challenging ligand to displace, however, which we have found requires harsh conditions to drive the substitution. The *E*_1/2_ of [Ru­(NH_3_)]^3+/2+^ in MeCN is 0.291 V vs Fc^+/0^, shown in Figure S12, and thus not consistent with the catalytic wave or quasi-reversible waves seen at fast scan rates and low NH_3_ concentrations. We therefore tracked the fate of the reaction using the one-electron oxidized putative catalyst [Ru­(Cl)]^2+^ and NH_3_ using NMR spectroscopy. [Ru­(Cl)]^2+^ was dissolved in MeCN-*d*_3_ in an NMR tube and ^15^NH_3_ was used as an ammonia source to track the nitrogen flow by ^15^N NMR spectroscopy; low temperatures were employed to slow the reaction rate and attempt to observe possible intermediates in addition to products. The samples were cooled to –40 °C, and ^15^NH_3_ was vacuum transferred to the NMR sample. Figure 2 shows that ^1^H NMR spectra of the products in addition to authentic samples of [Ru­(Cl)]^2+^ and [Ru­(Cl)]^+^. The initial spectra taken at –40 °C exhibits broad, ill-defined peaks, significantly shifted from both the [Ru­(Cl)]^2+^ and [Ru­(Cl)]^+^ spectra. This observation is consistent with electron self-exchange indicating the presence of both Ru^II^ and Ru^III^ oxidation states present in solution.^5^ When the NMR tube was warmed to 25 °C, the ^1^H NMR spectrum of the mixture was well matched to the spectrum [Ru­(Cl)]^+^ which indicates the clean reduction of [Ru­(Cl)]^2+^ to [Ru­(Cl)]^+^, with no detectable side products. This result clearly shows that [Ru­(Cl)]^+^ is the catalytic reaction cycle product with no detectable formation of [Ru­(NH_3_)]^2+^. This finding is in agreement with the EC′ mechanism applied to interpret the electrocatalysis measurements, but is surprising, contradicts prior reports,^12,26^ and demonstrates that the NH_3_ oxidation reaction proceeds through a different mechanistic pathway than previously reported for [Ru­(NH_3_)]^2+^.^5^

**Figure 2:**
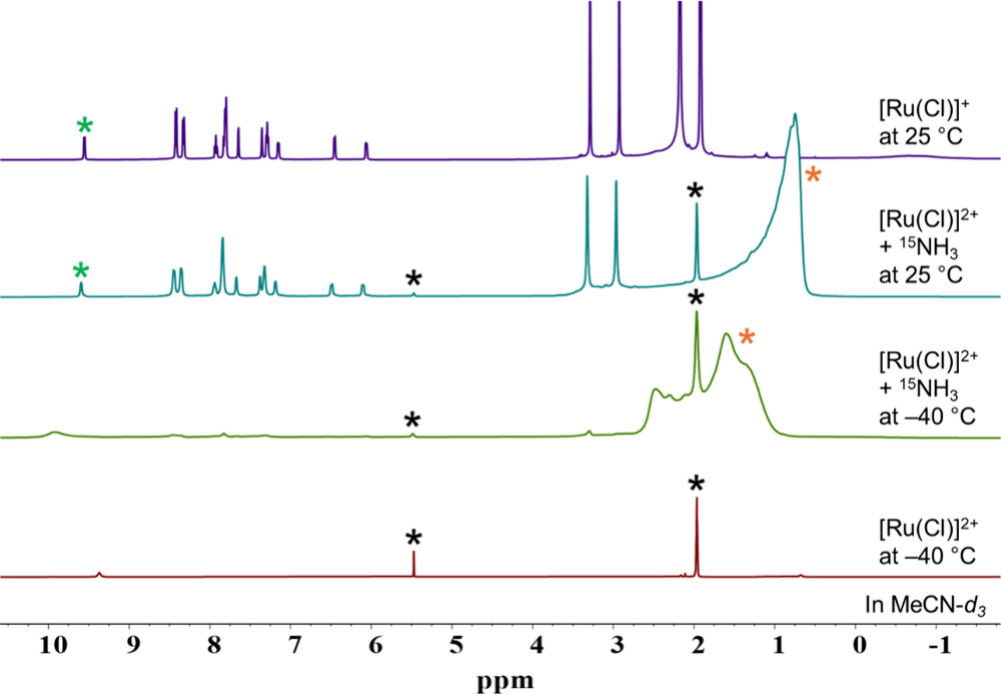
^1^H NMR spectra of 50 mM [Ru­(Cl)]^2+^ at –40 °C (red), adding excess ^15^NH_3_ to the 50 mM [Ru­(Cl)]^2+^ at –40 °C (dark green), the reaction mixture warmed to 25 °C (teal), [Ru­(Cl)]^+^ at 25 °C for comparison (purple). Asterisks indicate distinct diagnostic peaks for [Ru­(Cl)]^+^ (green), solvent peaks from DCM at 5.49 ppm (black), MeCN-*d*_3_ at 1.97 ppm (black), and ^15^NH_3_ (orange).

In order to track the fate of the presumed reductant, ^15^N NMR spectroscopy measurements were also performed for the same samples and conditions described above for ^1^H NMR spectroscopy. Three peaks are found in the ^15^N NMR spectra following the addition of ^15^NH_3_ to [Ru­(Cl)]^2+^ in MeCN-*d*_3_ at –40 °C, shown in Figure S14. These three peaks remain, and no other species are found when the sample is warmed up to 25 °C, shown in Figure 3. The peak at −136.93 ppm is assigned to the natural existence of ^15^N from MeCN-*d*_3_. The chemical shift suggests that the peak at 380.31 ppm is ^15^NH_3_.^27^ Further discussions about the resonance of ^15^NH_3_ in MeCN-*d*_3_ are shown in SI section (Figures S15 – S18). A singlet is found at −70.74 ppm regardless of whether ^1^H–^15^N NMR is coupled or decoupled, which is assigned to ^15^N_2_.^5^ Thus, the ^15^N NMR spectroscopy results indicate that NH_3_ is oxidized by [Ru­(Cl)]^2+^ to form N_2_.

**Figure 3:**
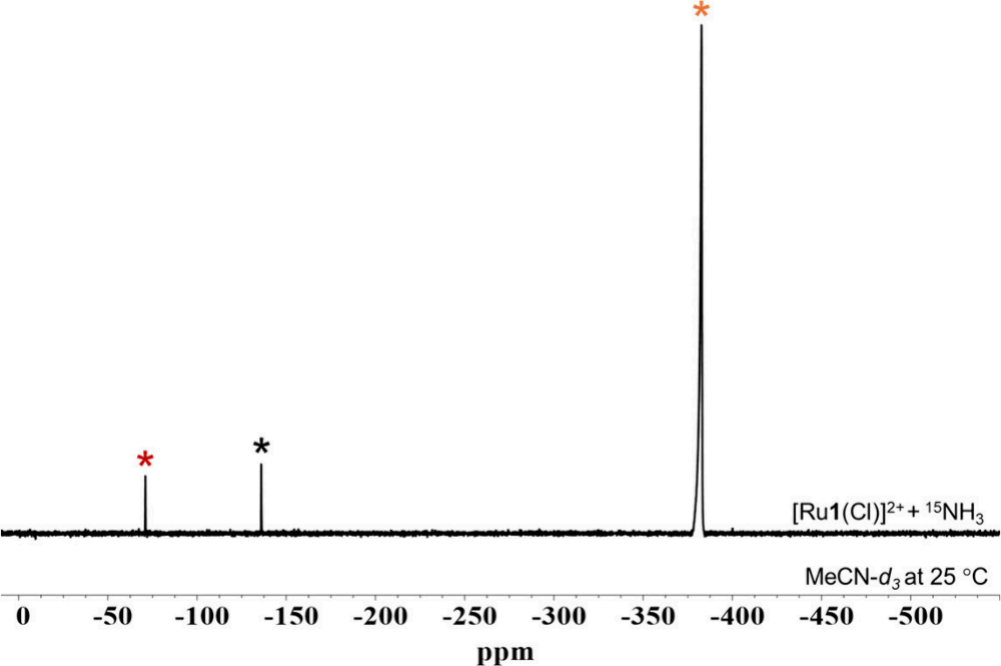
^15^N NMR spectra of the reaction mixture upon warmed up to 25 °C after adding excess ^15^NH_3_ to 50 mM [Ru­(Cl)]^2+^ at –40 °C with the ^1^H–^15^N decoupling on. The asterisks indicate diagnostic peaks of the natural abundance of ^15^N in the MeCN-*d*_3_ (black), ^15^NH_3_ (orange), and free ^15^N_2_ (red).

The reaction of [Ru­(Cl)]^2+^ with NH_3_ is surprisingly clean and does not form any [Ru­(NH_3_)]^2+^ as a side product when carried out in MeCN-*d*_3_. We note that magic blue has been shown to be inert to ammonia oxidation.^28^ No hydrazine was detected in ^1^H or ^15^N NMR spectroscopy measurements, or other intermediate species, which has been reported for similar ruthenium complexes employing pyridylpyrole ligands.^29^ While the NMR results cannot completely rule out insertion of NH_3_ in the bpy or tpy ligand frameworks as others have shown for Ru catalysts,^24,30^ those reports do not follow the EC′ electrocatalytic behavior seen here, indicating different, more complicated mechanisms. In addition, our prior report showed that catalysis by [Ru­(NH_3_)]^2+^ produced intermediate dinitrogen species that are displaced by solvent to form [Ru­(NCMe)]^2+^ which is inert to substitution by NH_3_. The lack of bridging dinitrogen complexes or [Ru­(NCMe)]^2+^ in the NMR spectra also supports an alternative mechanism than our prior reports.

Taken together, the results suggest the AOR is triggered by a one-electron oxidation reaction. The Manthiram group recently reported the heterogeneous ammonia oxidation reaction in MeCN occurs by an outersphere electron transfer reaction to form a radical cation intermedate.^31^ Calculations show that electron transfer to an ammonia trimer is a lower energy pathway than isolated ammonia molecule or a dimer, which would follow a third order pathway in ammonia concentration.^31,32^ The reaction order was found to be ∼ 3 in ammonia concentration, in excellent agreement with the report by Warren using ferrocenium to drive the AOR, consistent with the lower energy outersphere electron transfer to ammonia trimers. Oxidation of dimers and trimers by ferrocenium is nominally isoenergetic,^29^ thus the more positive [Ru­(Cl)]^2+^ is thermodynamically competent to drive the AOR. We therefore hypothesize that AOR via [Ru­(Cl)]^+^ is triggered by a one-electron oxidation of ammonia species in solution. Our group is actively investigating the mechanistic details, scope and limitations of this proposed pathway of AOR. The implications of this ongoing work are important not only in homogeneous catalyst design, but new strategies to control and improve heterogeneous catalysis.

## Supplementary Material



## Notes

Additional synthesis details and procedures; NMR spectra of all products; Electrochemical methods and characterization data; Procedure for stoichiometric reactions monitored with NMR spectroscopy; NMR spectra and analysis of ^15^NH_3_ in MeCN-*d*_3_ (PDF)
