# Supplementary material for: Electrocatalytic Ammonia Oxidation with Coordinatively Saturated Ruthenium Catalyst
Source: Inorg Chem. 2025 Jul 1;64(27):13599–603. doi: 10.1021/acs.inorgchem.5c02418 (PMC12264961; doi:10.1021/acs.inorgchem.5c02418)
Supplement: Supplementary file 1 [file ic5c02418_si_001.pdf]

Supporting Information

Electrocatalytic Ammonia Oxidation with Coordinatively Saturated Ruthenium Catalyst

Chuan-Pin Chen[a,†], Oluwafemi Abubakar[a,†], Xiaoyin Zhang[a] and Thomas W. Hamann[a,*]

[†]Authors contributed equally to this work

a Department of Chemistry, Michigan State University, 578 S Shaw Ln, East Lansing, MI, 48824 USA

* hamann@msu.edu

Synthesis Details

All syntheses were performed under a dry, oxygen-free nitrogen atmosphere using standard Schlenk techniques or in a glove box. Acetonitrile (MeCN) and dichloromethane (DCM) were dried by activated molecular sieves under nitrogen. Terpyridine (tpy) and 4,4′-bis(dimethylamino)-2,2′-bipyridine (dmabpy) were purchased from Ambeed Chemicals and used as received. NH_4OTf was purchased from Sigma-Aldrich or Oakwood Chemical and recrystallized twice by THF/DCM or THF/chloroform before electrochemistry measurements. Other chemical reagents were purchased from commercial sources and used as received.

Anhydrous NH_3 in a cylinder was obtained from Airgas and dried through two columns filled with barium oxides. 1 L of $^{15}NH_3$ in a 450 mL carbon steel lecture bottle with stainless steel CGA 110/180 valve was obtained from Sigma-Aldrich. Since its pressure is only slightly above atmospheric pressure, care must be taken when extracting $^{15}NH_3$ from the cylinder through vacuum transfer.

Column chromatography was performed on 240 - 400 mesh Silica P-Flash silica gel, and basic alumina obtained from Sigma. Thin-layer chromatography was performed on 0.25 mm thick aluminum–backed silica gel plates and visualized with ultraviolet light (λ = 254 nm).

^{1}H, ^{13}C, and ^{19}F NMR spectra were recorded on a Varian 500 MHz DD2 Spectrometer equipped with a ^{1}H-^{19}F 5 mm Pulsed Field Gradient (PFG) Probe. Spectra were taken in $CDCl_3$ referenced to 7.26 ppm in 1H NMR and 77.0 ppm in ^{13}C NMR. ^{15}N NMR spectra were referenced to CH_3NO_2 = 0 ppm. All coupling constants are apparent J values measured at the indicated field strengths in Hertz (s = singlet, d = doublet, t = triplet, q = quartet, dd = doublet of doublets, ddd = doublet of doublet of doublets, bs = broad singlet). NMR spectra were processed for display using the MNova software program with only exponential, phase correction, baseline corrections, and zero filing applied.

Synthesis of [Ru(tpy)(dmabpy)Cl](PF$_6$), [Ru(Cl)]$^+$

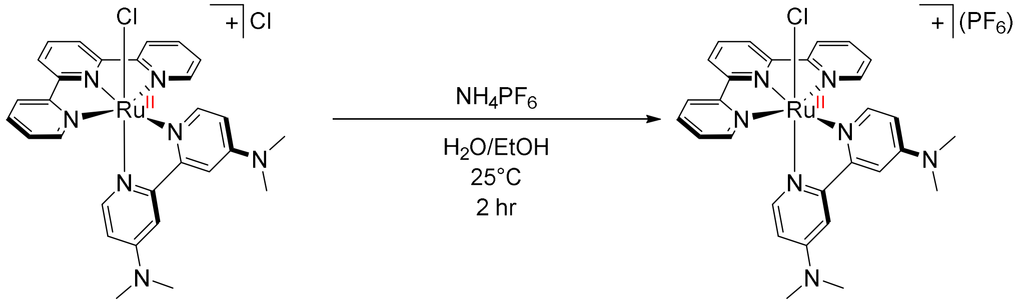

[Ru^{II}(tpy)(dmabpy)(Cl)]Cl (120.0 mg, 0.1853 mmol), water (15 mL), ethanol (5 mL), and NH_4PF_6 (120.0 mg, 0.7362 mmol) were added into a single-necked flask. After gently stirring for 2 hours, this solution was filtered off. The residue is washed with a small amount of water, and then ether. The solids were collected and dried under 80°C for 2 hours to obtain 89.5mg of product. (63% yield)

^{1}H NMR (500 MHz, MeCN-*d$_3$*): δ 9.60 (d, *J* = 6.7 Hz, 1H), 8.46 (d, *J* = 8.1 Hz, 2H), 8.37 (d, *J* = 7.9 Hz, 2H), 7.97 (t, *J* = 8.1 Hz, 1H), 7.89 – 7.82 (m, 4H), 7.69 (d, *J* = 2.9 Hz, 1H), 7.39 (d, *J* = 2.9 Hz, 1H), 7.33 (t, *J* = 7.2 Hz, 2H), 7.20 (dd, *J* = 6.8, 2.8 Hz, 1H), 6.50 (d, *J* = 6.8 Hz, 1H), 6.10 (dd, *J* = 6.9, 2.9 Hz, 1H), 3.33 (s, 6H), 2.97 (s, 6H), (figure S1).
^{19}F NMR (470 MHz, MeCN-*d$_3$*): δ-72.95 (d, *J* = 706.41, 6F), (figure S2).

Synthesis of [Ru^{III}(tpy)(dmabpy)Cl](PF$_6$)$_2$, [Ru(Cl)]$^{2+}$

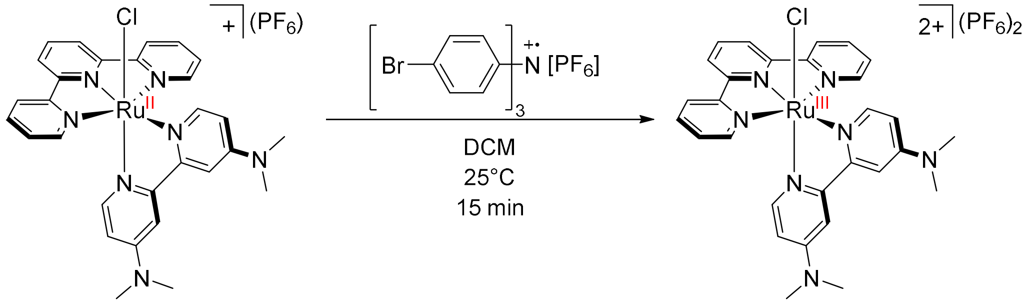

This compound was synthesized using a method previously reported.[1] Briefly, [NAr$_3$][PF$_6$] (89.6 mg, 0.143 mmol, 1.16 equiv) in DCM was slowly added via pipette to a vial containing [Ru(tpy)(dmabpy)Cl](PF$_6$) (95.8 mg, 0.123 mmol) in DCM and stirred for 15 min in a glove box. The solution was filtered, and the solids were washed with DCM several times to obtain a green product (101.5 mg, 88%).

^{1}H NMR spectroscopy (500 MHz, MeCN-d_3): δ 56.18, 53.54 (d, 6H, see notes), 28.87 (bs, 2H), 14.65 (br, 6H, see notes), 12.68 (bs, 2H), 9.37 (bs, 2H), 0.68 (bs, 1H), -2.44(bs, 1H), -10.89 (bs, 1H), -11.44 (bs, 2H), -14.50 (bs, 2H), -16.07 (bs, 1H), -20.52 (bs, 1H), -24.44 (bs, 1H), -30.88 (bs, 1H), (figure S3).
Elemental analysis: found (calculated): C, 38.16 (38.61); H, 3.22 (3.24); N, 10.58 (10.87).

^{15}N NMR spectra show only a single peak corresponding to the natural abundance of ^{15}N in the solvent. No peak attributable to $[Ru(Cl)]^{2+}$ is detected, which is consistent with the behavior of complexes containing paramagnetic metal centers (figure S4).

ESI-MS analysis: Calc. for $C_{29}H_{29}ClF_6N_7PRu$ $[M-Cl][PF_6]^+$ ($[Ru^{III}(Cl)][PF_6]^+$) and $C_{29}H_{29}ClN_7Ru$ $[M-Cl]^{2+}$ ($[Ru^{III}(Cl)]^{2+}$) *m/z* 757.09 and 612.12. Found *m/z* 757.0886 and 612.1225 (figure S6).

Preparation of Dried MeCN-d_3

The molecular sieves were placed in a Schlenk flask and dried under vacuum overnight at ~250 °C to activate them, then transferred to the glovebox. Next, a portion of the sieves was added to MeCN-d_3 to dry, and this solution was left overnight, which is called “pre-dried” MeCN-d_3. In a separate vial, activated molecular sieves were added and rinsed three times with "pre-dried" MeCN-d_3 to remove any debris. The remaining "pre-dried" MeCN-d_3 was then carefully poured into the vial containing the “pre-rinsed” activated sieves to obtain dried MeCN-d_3, which should not show a water peak in the NMR measurements. It is worth mentioning that MeCN-d_3 should not be stored with molecular sieves for too long (roughly 2~3 weeks) because impurities showing two broad triplets at 6.07 and 5.50 ppm will appear, even though the exact reason is unclear. The accurate time for forming the impurity is uncertain. It has been observed that washing debris will be helpful to keep it in good shape for a longer time.

NMR Spectra

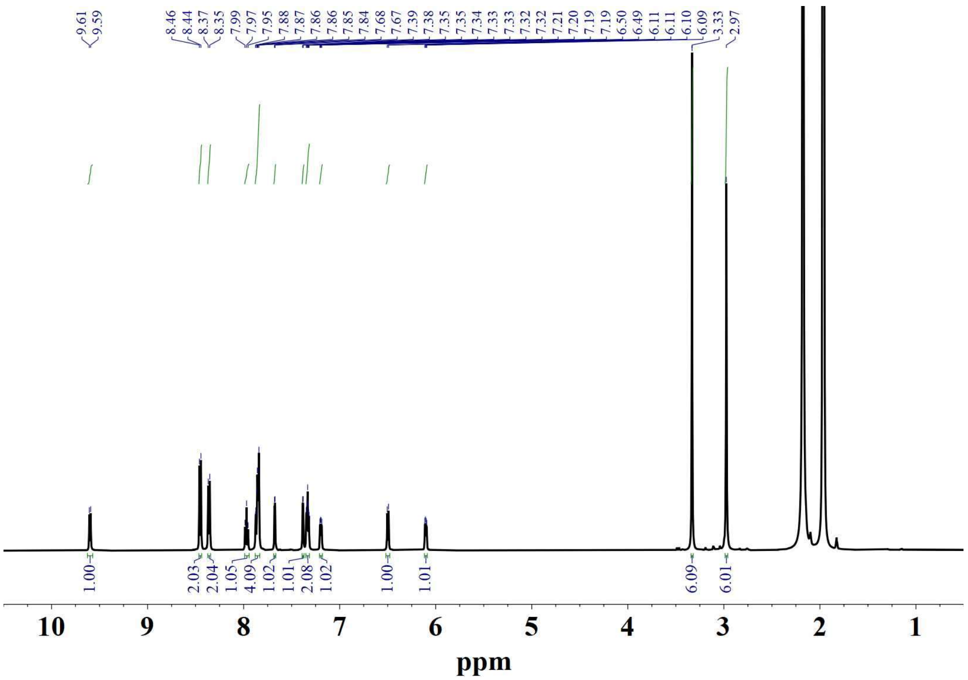

Figure S1. ^{1}H NMR spectrum of $[Ru(Cl)](PF_6)$ in MeCN-d_3.

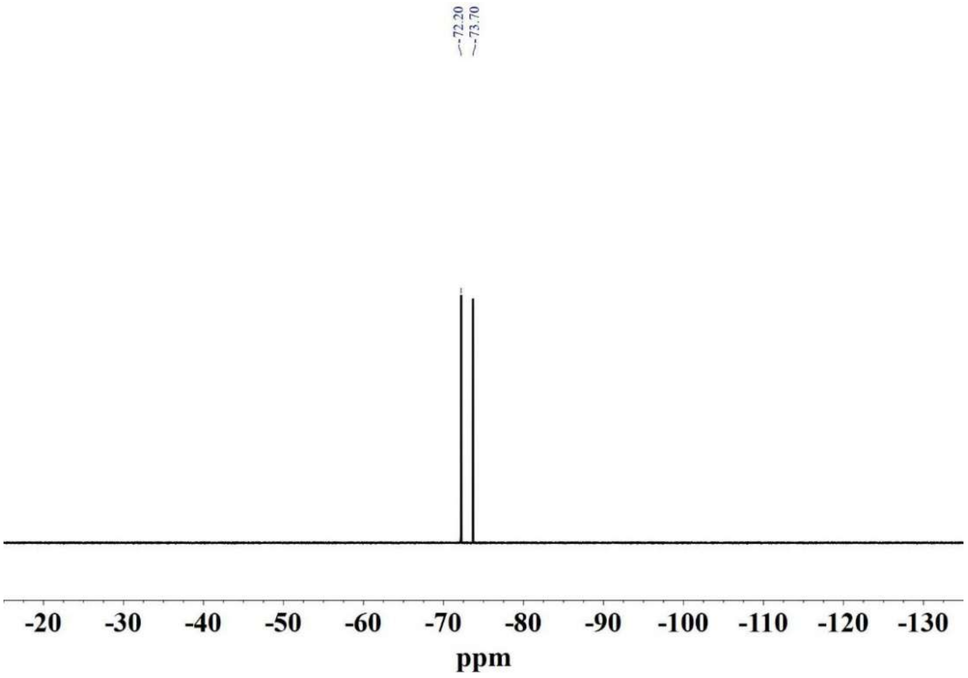

Figure S2. ^{19}F NMR spectrum of $[Ru(Cl)](PF_6)_2$ in MeCN-d_3.

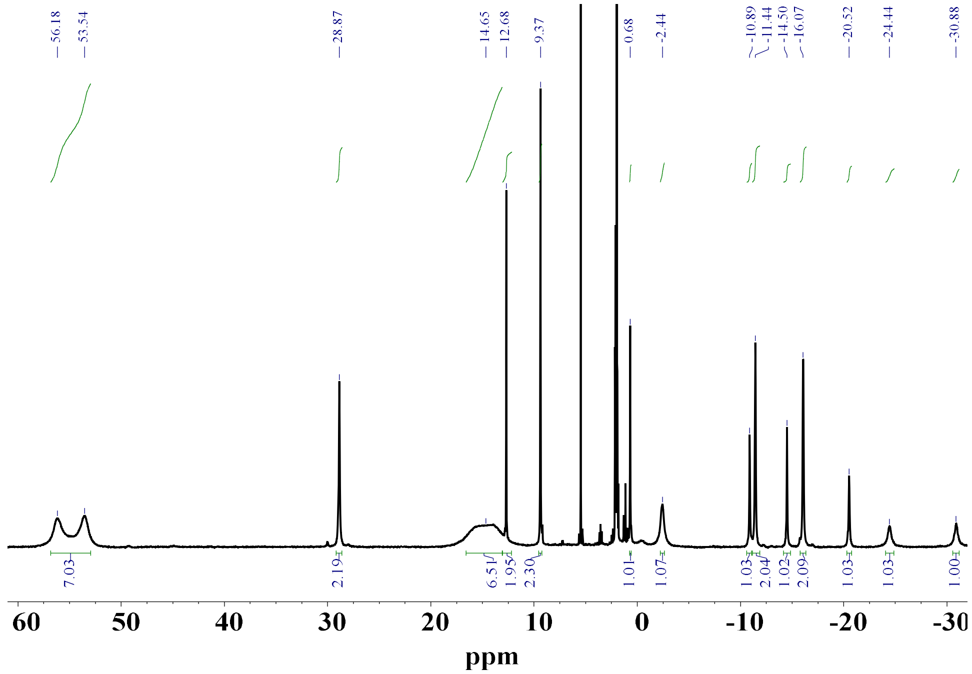

Figure S3. ^{1}H NMR spectrum of $[Ru(Cl)](PF_6)_2$ in MeCN-d_3.

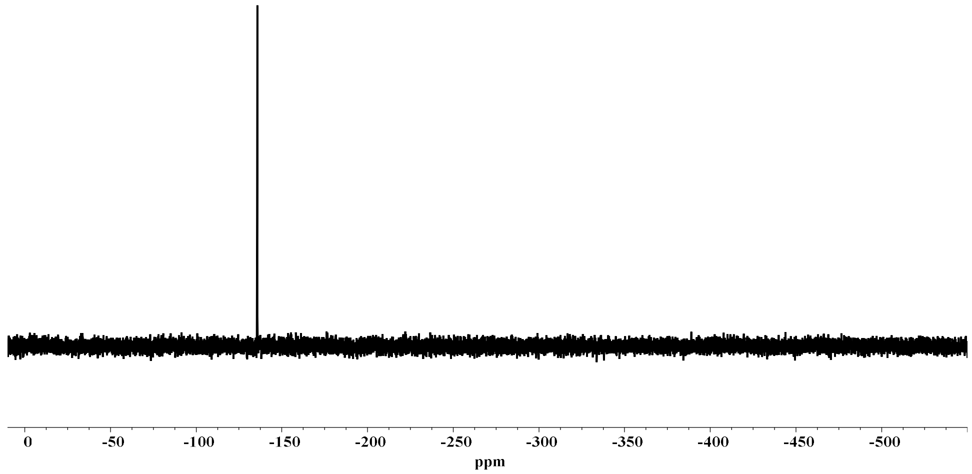

Figure S4. ^{15}N NMR spectrum of $[Ru(Cl)](PF_6)_2$ in MeCN-d_3. The only single peak corresponds to the natural abundance of ^{15}N in the solvent.

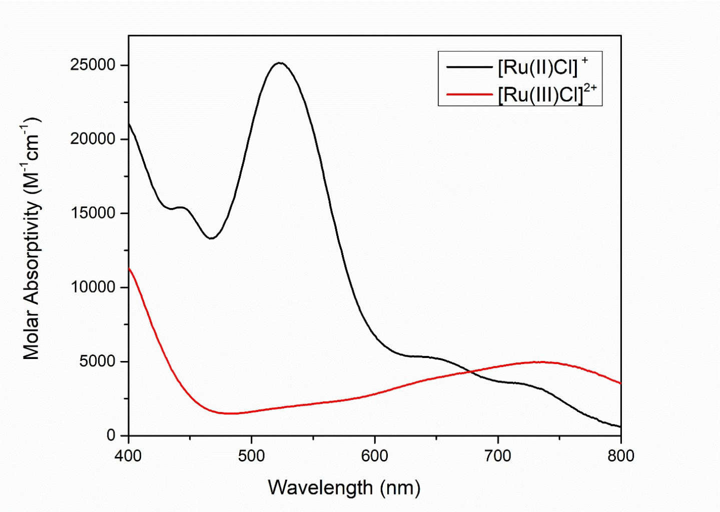

Figure S5. UV-Vis spectra of [Ru(Cl)](PF_6) and [Ru(Cl)](PF_6)$_2$. The loss of the MLCT band at 525 nm shows complete conversion of Ru^{II} to Ru^{III}.

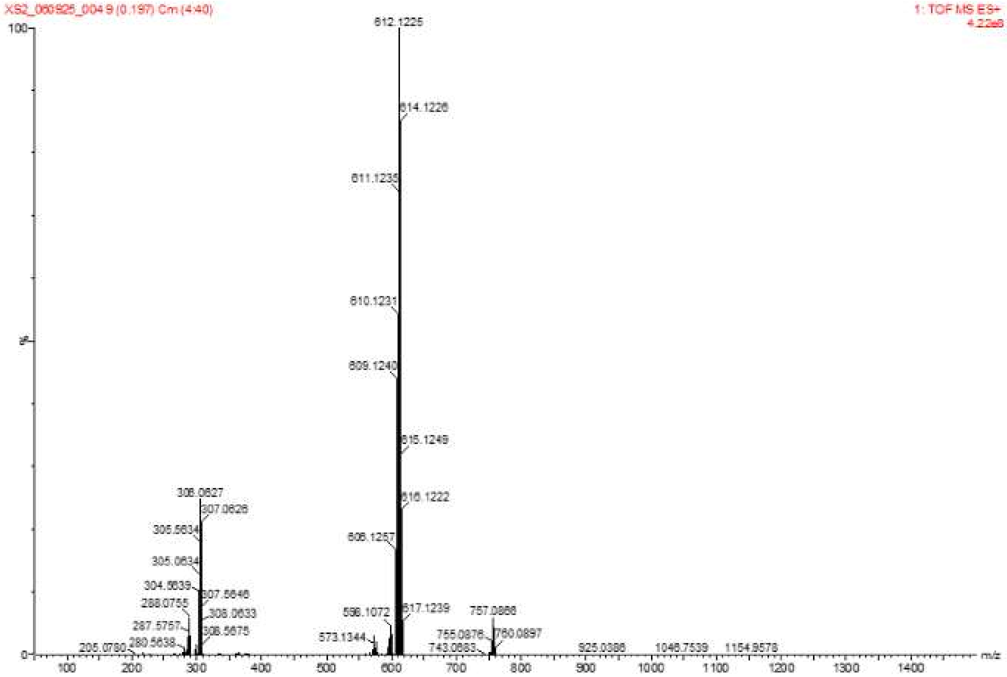

Figure S6. The ESI-MS analysis of [Ru(Cl)](PF_6)$_2$. The calculated *m/z* for $C_{29}H_{29}ClF_6N_7PRu$ ([Ru^{III}(Cl)][PF_6]$^+$) and $C_{29}H_{29}ClN_7Ru$ ([Ru^{III}(Cl)]$^{2+}$) are 757.09 and 612.12.

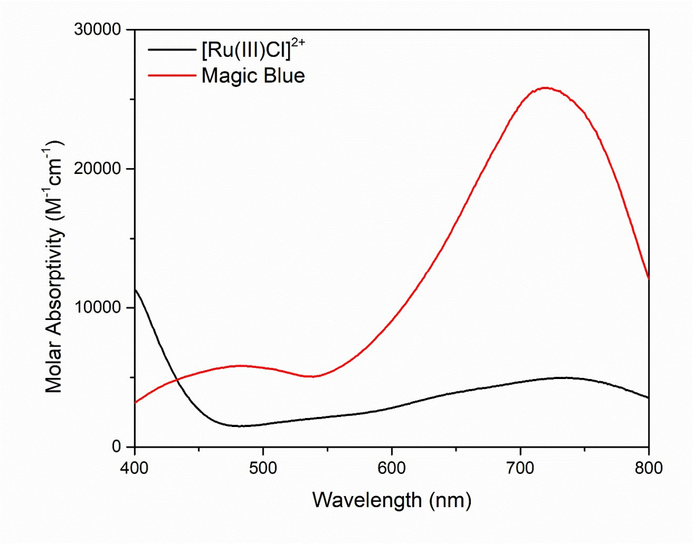

Figure S7. Absorption spectra of [Ru(Cl)]$(PF_6)_2$ and magic blue for comparison.

Electrochemistry Measurements

The electrochemical measurements were carried out with an Autolab potentiostat (PGSTAT128N) equipped with Nova electrochemical software. The electrolyte was purged with argon for 3 minutes to remove any residual oxygen prior to cyclic voltammetry measurements. The electrolyte was purged with anhydrous NH_3 through a stainless-steel needle for 15 min to reach a saturated NH_3 solution prior to electrocatalysis measurements. The potential of the reference electrode was measured vs standard ferrocene/ferrocenium couple in MeCN and was found to be 0.36 V vs Ag/AgCl. The concentration of saturated ammonia in acetonitrile was determined by following previously published procedure and was determined to be 1.32 M.[2] Other concentrations were obtained by diluting the saturated solution using a solution of known concentration of the catalyst and supporting electrolyte in acetonitrile.

Diffusion Coefficient

The diffusion coefficient of was determined using the Randles-Sevcik equation.[3] From the cyclic voltammogram, a plot of the square root of the scan rate against the peak current was carried out and the diffusion coefficient determined using the formula;

$$I_p = 0.4463\sqrt{\frac{nF}{RT}}n\mathrm{FA}[\mathrm{C_0}]\sqrt{Dv}$$

Where, I_p is the peak current, F is Faraday's constant (F = 96485 C mol^{-1}), R is the universal gas constant (R = 8.314 J K^{-1} mol^{-1}), T is temperature (T = 300 K), n is the number of electrons transferred, A is the

active surface area of the electrode, D is the diffusion coefficient of the complex, $[C_0]$ is the concentration of the catalyst, and ν is the scan rate (V/s).

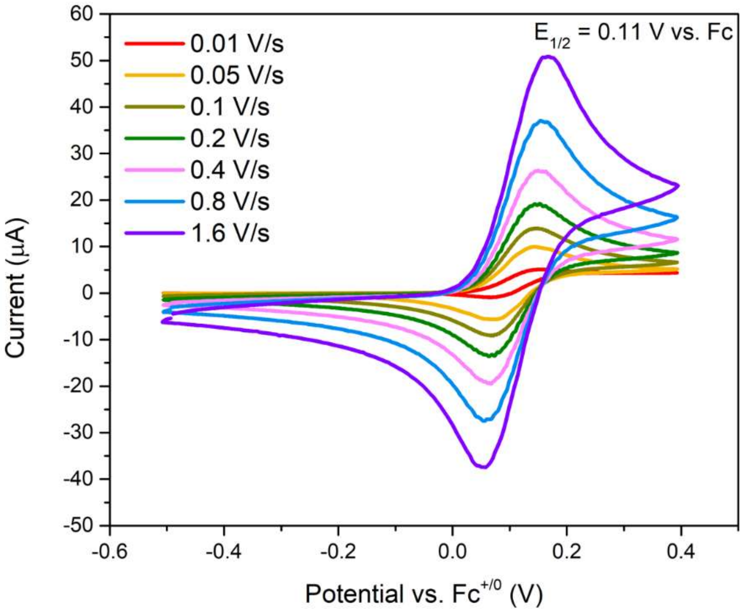

Figure S8. Cyclic voltammograms of 2.5 mM $[Ru(Cl)]^+$ at different scan rates in 0.1 M NH_4OTf MeCN solution.

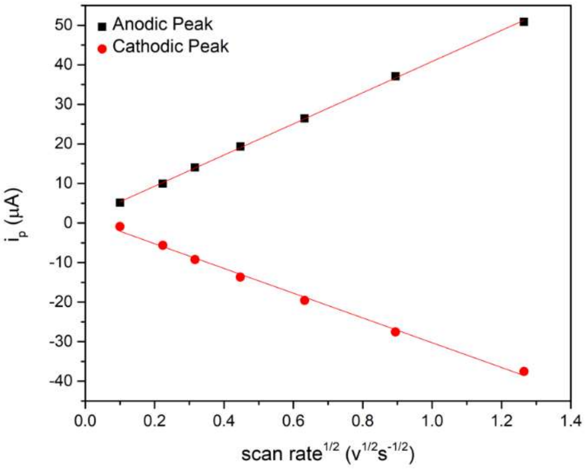

Figure S9. Plot of the square root of the scan rate against the peak current for $[Ru(Cl)]^+$. The slope was 3.94×10^{-5} A $V^{1/2}$ (Anodic Peak) and -3.14×10^{-5}A $V^{1/2}$ (Cathodic Peak). Diffusion coefficients of 3.48×10^{-6} cm^2/s (anodic) and 2.21×10^{-6} cm^2/s (cathodic) were determined from the slope using the Randles-Sevcik equation.

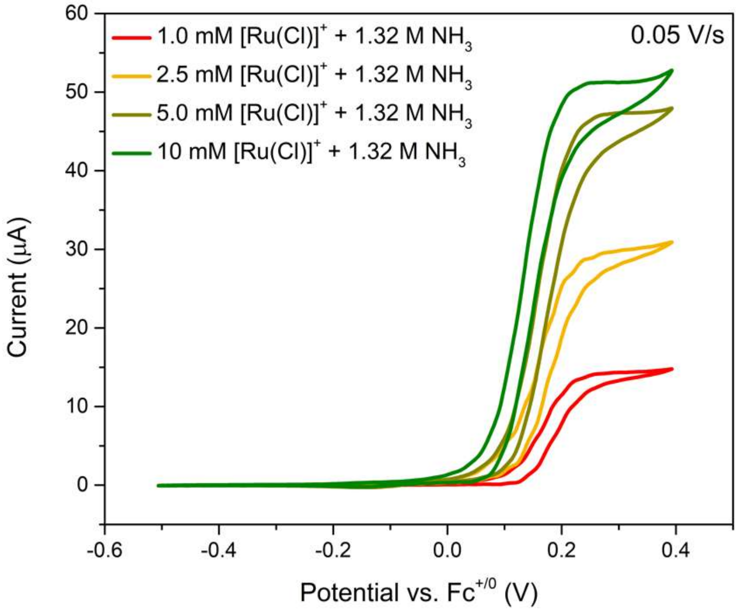

Figure S10. Cyclic Voltammograms of 1.0 mM (red), 2.5 mM (orange), 5.0 mM (dark yellow), and 10 mM (green) of $[Ru(Cl)]^+$ with saturated NH_3 in 0.1 M NH_4OTf MeCN solution at scan rate 0.05 V/s.

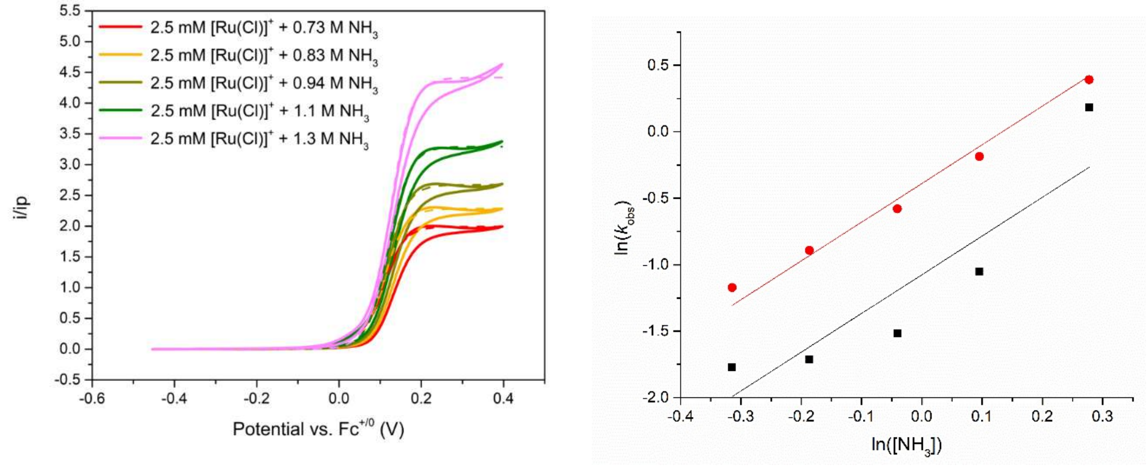

Figure S11. Left: Cyclic Voltammograms of 2.5 mM $[Ru(Cl)]^{+}$ with varying concentrations of NH_3 in MeCN solutions containing 0.1 M NH_4OTf supporting electrolyte at a scan rate 0.01 V/s. Currents are normalized to the anodic peak current of $[Ru(Cl)]^{+}$ in the absence of catalysis. Right: Plot of 2 independent measurements of $\ln(k_{obs})$ vs $\ln([NH_3])$ fitted to a straight line with a common slope.

The reaction order, α, in $[NH_3]$ is given by the slope: $k_{obs} = k_c[NH_3]^{\alpha} \rightarrow \ln(k_{obs}) = \ln(k_c) + \alpha\ln([NH_3])$. The slope was found to be 2.9 ± 0.4.

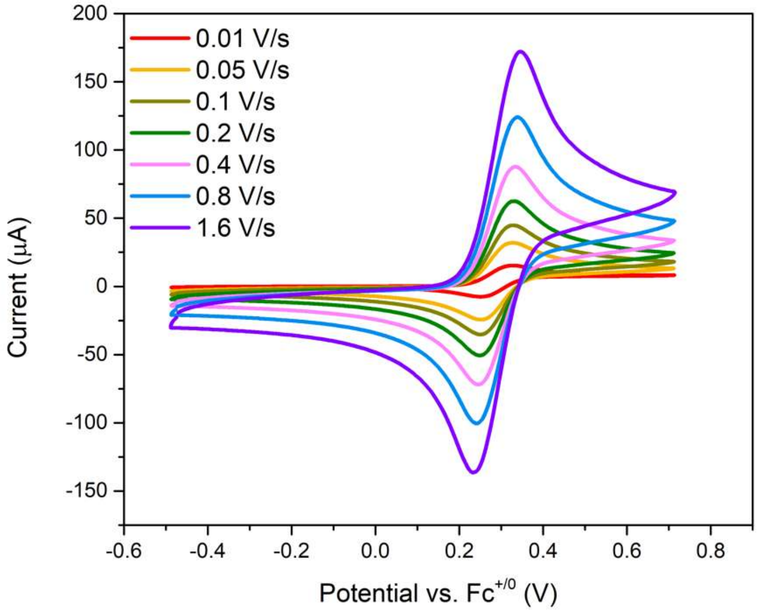

Figure S12. Cyclic voltammograms of 2.5 mM $[Ru(NH_3)]^{2+}$ in MeCN solutions containing 0.1 M NH_4OTf supporting electrolyte at different scan rates.

Stoichiometric NMR Spectroscopy Measurements for Adding $^{15}NH_3$ to Ru^{III} Complex

In the glovebox, Ru^{III} complex, $[Ru(Cl)]^{2+}$, was dissolved by dried MeCN-d_3 in a vial, and then this solution was carefully transferred to J. Young NMR tube by syringe. After sealed, this NMR sample was taken out from glovebox, connected to Schlenk line (Vac/nitrogen 3 times for tubing), and "slowly" cooled down to ~ –40 °C by MeCN/dried ice cooling bath until solution was frozen. Next, the sample was subject to vacuum and then vacuum transfer is performed to condense $^{15}NH_3$ from commercial $^{15}NH_3$ gas cylinder to NMR tube. This sample was maintained to –40 °C and immediately transferred to NMR probe for VT NMR measurements, including ^{1}H, ^{1}H-^{15}N decoupled, and ^{1}H-^{15}N coupled spectra (figure 2, figure 3, figure S10).

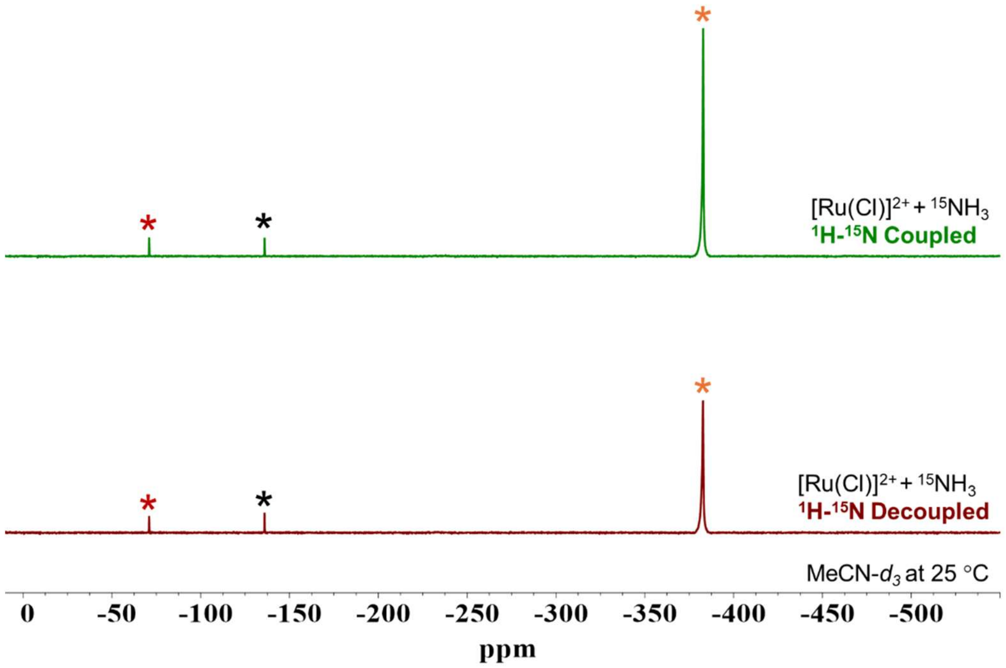

Figure S13. The ^{15}N NMR spectra of reaction mixture upon warming up to 25 °C after adding $^{15}NH_3$ to $[Ru(Cl)]^{2+}$ at –40 °C with the proton decoupling on (red) and when the proton decoupling is off (green). Asterisks indicate distinct diagnostic peaks for free $^{15}N_2$ (red), natural abundance of ^{15}N in the MeCN-d_3 (black), and $^{15}NH_3$ (orange).

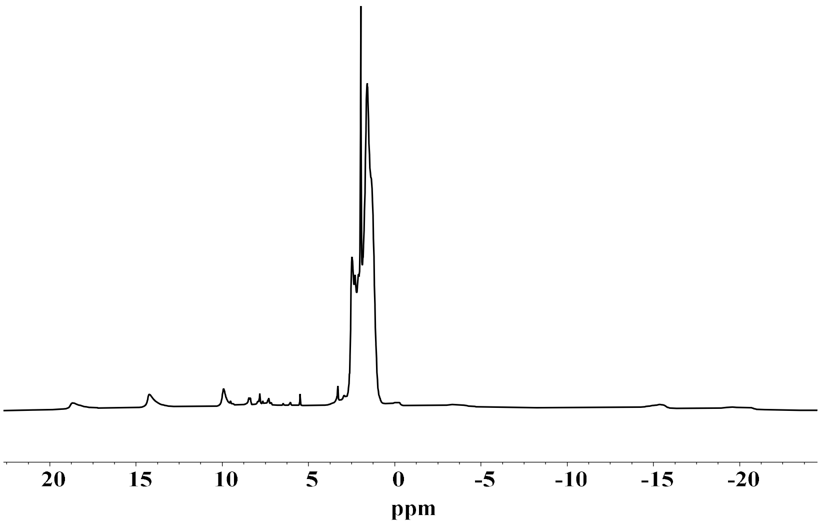

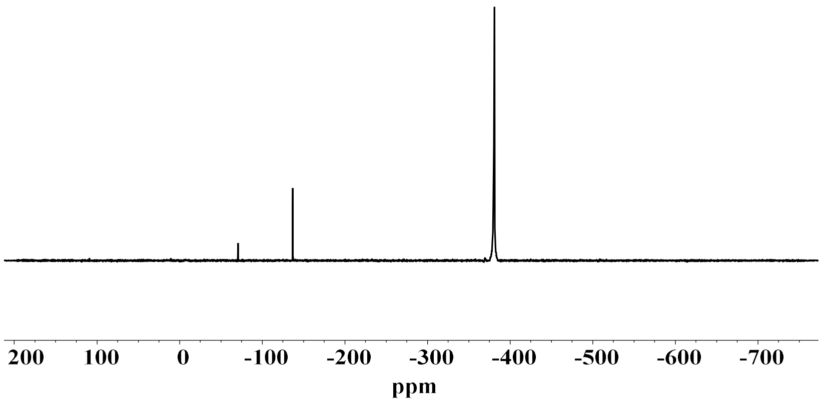

Figure S14. Top: ^{1}H NMR spectrum of reaction mixture upon after adding $^{15}NH_3$ to $[Ru(Cl)]^{2+}$ at –40 °C. Bottom: ^{15}N NMR spectra of the same reaction mixture upon after adding $^{15}NH_3$ to $[Ru(Cl)]^{2+}$ at –40 °C (proton decoupling off).

Peak Analysis for $^{15}NH_3$ in MeCN-d_3

When analyzing the spectra of adding $^{15}NH_3$ to $[Ru(Cl)]^{2+}$, the peak from $^{15}NH_3$ at 380.31 ppm is a singlet as expected when the ^{1}H-^{15}N is decoupled, surprisingly however, this peak does not split when the ^{1}H-^{15}N decoupler is off (figure S10). The presence of 3 adjacent protons on the ^{15}N should produce a quartet for this resonance. Similar cases of influenced peaks have been previously reported for ^{15}N NMR spectra of L-glutamine enriched with ^{15}N in an amide position with water.[4] The lack of ^{1}H-^{15}N coupling observed for $^{15}NH_3$ might be caused by the exchange of protons on ^{15}N that affects the ^{1}H-^{15}N interaction. In order to confirm our assignment, a control experiment was conducted by adding $^{15}NH_3$ to MeCN-d_3 and measured it at –40 °C, a temperature chosen to minimize the rate of proton exchange. The ^{1}H NMR spectrum shows the resonance for NH_3 is broad, implying the rapid exchange of protons (figure S11).[5] The ^{15}N NMR spectrum shows a singlet at 380 ppm when the ^{1}H-^{15}N is de-coupled (figure S12) or coupled (figure S13), consistent with our assignment and observation for the reaction mixture containing $^{15}NH_3$. In addition, ^{1}H-^{15}N HSQC further confirmed that the ^{1}H-^{15}N interaction is affected, providing reasonable explanation for all observations (figure S14).

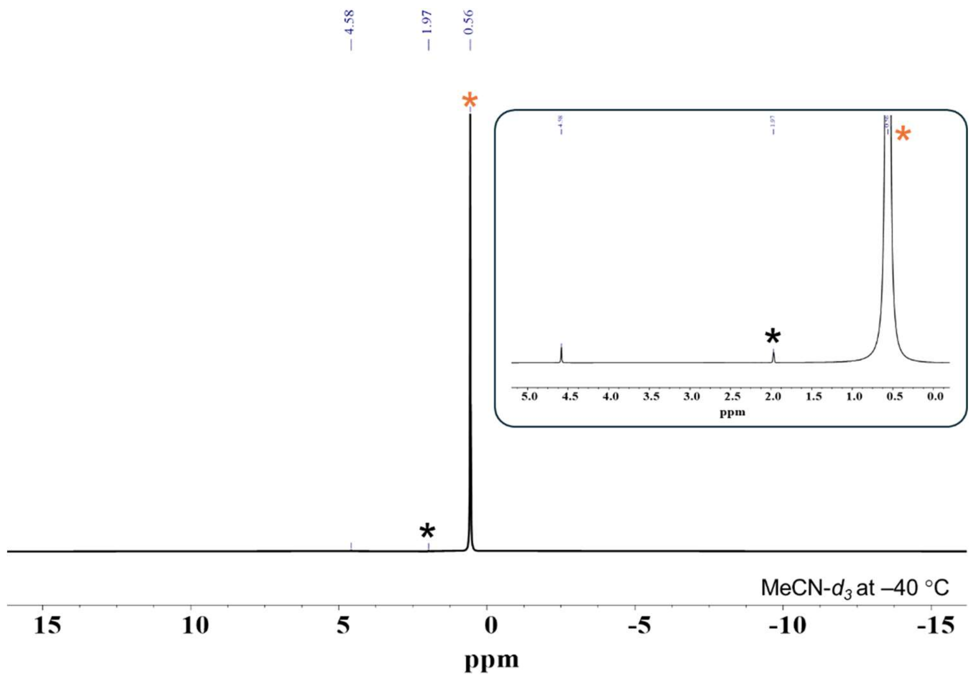

Figure S15. ^{1}H NMR spectrum for $^{15}NH_3$ in MeCN-d_3. The asterisks indicate distinct diagnostic peaks for the residue in MeCN-d_3 (black) and $^{15}NH_3$ (orange) at –40 °C.

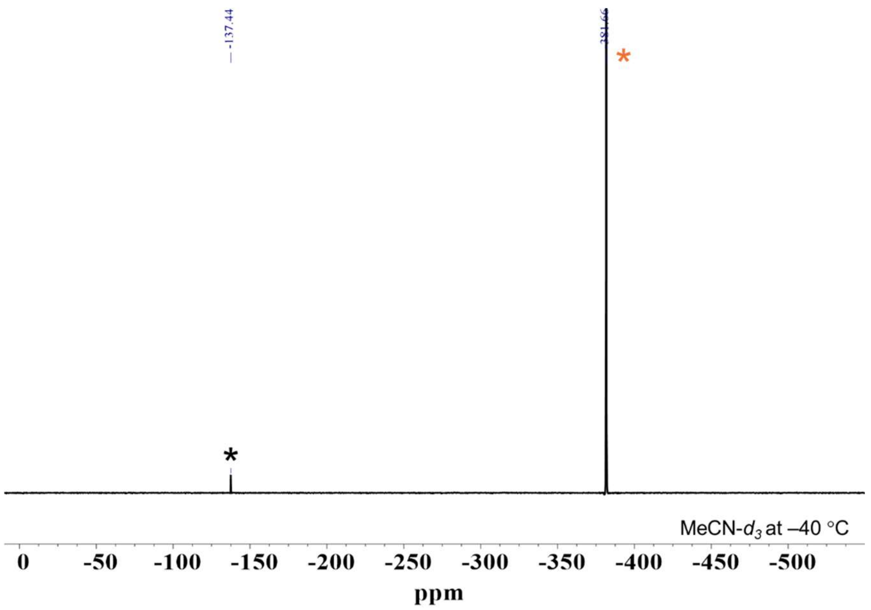

Figure S16. ^{15}N NMR spectrum with ^{1}H-^{15}N decoupled for $^{15}NH_3$ in MeCN-d_3. The asterisks indicate distinct diagnostic peaks for the natural abundance of ^{15}N in MeCN-d_3 (black) and $^{15}NH_3$ (orange) at –40 °C.

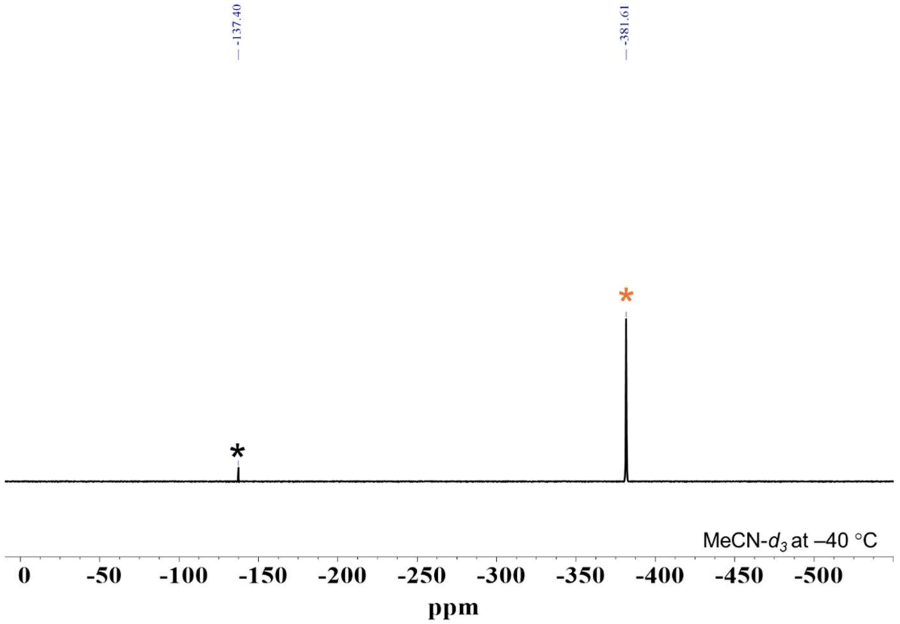

Figure S17. ^{15}N NMR spectrum with ^{1}H-^{15}N coupled for $^{15}NH_3$ in MeCN-d_3. The asterisks indicate distinct diagnostic peaks for the natural abundance of ^{15}N in MeCN-d_3 (black) and $^{15}NH_3$ (orange) at –40 °C.

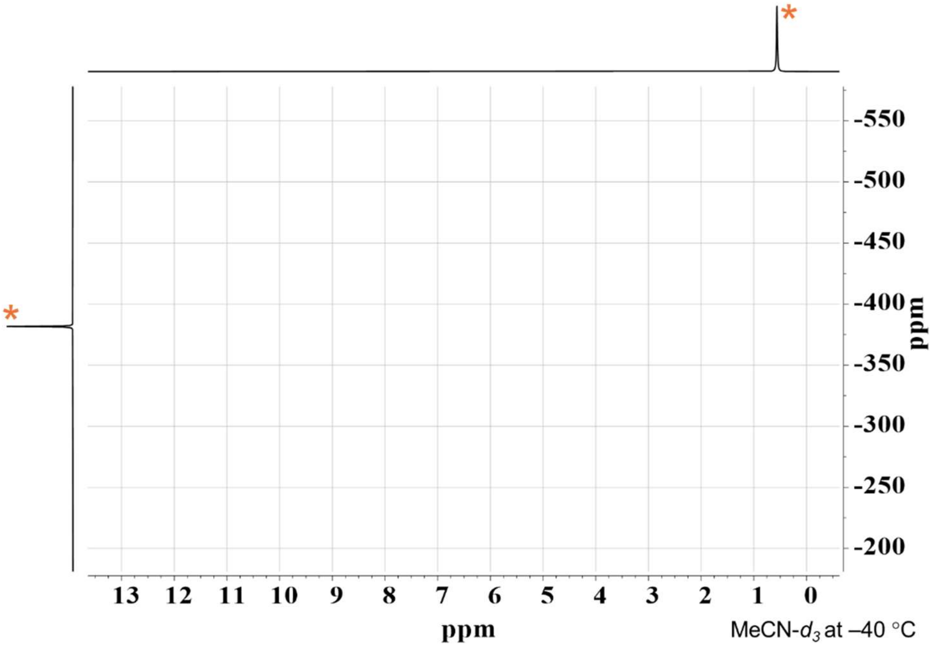

Figure S18. ^1H-^{15}N HSQC for $^{15}NH_3$ in MeCN-d_3. The orange asterisks indicate diagnostic peaks for $^{15}NH_3$ in ^1H NMR and ^{15}N NMR at –40 °C.

References

(1) Chen, C.-P.; Alharbi, W.; Cundari, T. R.; Hamann, T. W.; Smith, M. R., 3rd. Deciphering the Mechanism of Base-Triggered Conversion of Ammonia to Molecular Nitrogen and Methylamine to Cyanide. *J. Am. Chem. Soc.* **2023**, *145* (48), 26339-26349. DOI: 10.1021/jacs.3c09879.
(2) Ahmed, M. E.; Raghibi Boroujeni, M.; Ghosh, P.; Greene, C.; Kundu, S.; Bertke, J. A.; Warren, T. H. Electrocatalytic Ammonia Oxidation by a Low-Coordinate Copper Complex. *J. Am. Chem. Soc.* **2022**, *144* (46), 21136-21145. DOI: 10.1021/jacs.2c07977.
(3) Elgrishi, N.; Rountree, K. J.; McCarthy, B. D.; Rountree, E. S.; Eisenhart, T. T.; Dempsey, J. L. A Practical Beginner's Guide to Cyclic Voltammetry. *J. Chem. Educ.* **2018**, *95* (2), 197-206. DOI: 10.1021/acs.jchemed.7b00361.
(4) Blomberg, F.; Maurer, W.; Ruterjans, H. 15N nuclear magnetic resonance investigations on amino acids. *Proc. Natl. Acad. Sci. U.S.A.* **1976**, *73* (5), 1409-1413. DOI: 10.1073/pnas.73.5.1409.
(5) Ogg, R. A. Proton Magnetic Resonance Spectrum of Ammonia and the Interactions Involved in Hydrogen Bond Association. *J. Chem. Phys.* **1954**, *22* (3), 560-561. DOI: 10.1063/1.1740107.
